# Country Roads

**Published:** 1890-04-05

**Authors:** 


### The Hospital

A Weekly Journal of

Science, Medicine, Nursing, and Philanthropy.

*For Hospitals, Asylums, Medical Practitioners, Students, Nurses, Families, Parishes, Congregations, and the Charitable Public.*

Vol. VIII.—No. 184.] [April 5, 1890.

### COUNTRY ROADS.

Poor human nature! a very simple set of circumstances is sufficient to show it at its weakest if not at its worst. A recently published little pamphlet, which means but little to those who know nothing of country secrets, is to the man of country breeding and experience, quite pathetic reading. What can pathos have to do with country roads? Not much, perhaps, directly; but it can have a great deal to do with those human beings who use the roads, manage them, wrangle over them, pay highway rates, or refuse to pay them, and who generally make at least a considerable portion of their lives miserable about their highways. The gentleman and the farmer both like riding and driving on good, firm, and smooth roads; but whilst they both deeply enjoy the privilege they decidedly hate paying for it. Small wonder is it, after all, when the thing is fairly thought about; for neither the landlords nor the farmers are the exclusive users of the roads. Anybody who chooses to live in the country for a year or two and to watch the road traffic from day to day, will be surprised at the number of light carriages, cycles of all sorts, butchers' and other tradesmens' carts, waggons carrying stone, brick, or other building material, belonging in most cases either to very small ratepayers or to persons not ratepayers at all in the particular district. Yet, in spite of this vast traffic which oftentimes hardly concerns the landlord or the farmer even in the slightest degree, those two-class representatives have to bear almost the entire burden of the maintenance of the roads. When it is remembered that agricultural prices are now such as to reduce the tenant farmer to partial, and the landlord often to complete poverty, it is not surprising that the question of the maintenance of the country roads should be a constant cause of grief and malediction to country people.

This question has a "health" aspect; and it is that which makes it of importance to a certain class of town dwellers. It has, moreover, an important bearing on the progress or regress of civilization; and as the developmentarians are now anxious to take civilization under their special protection, we, as humble subalterns in the great "development" army, desire to perform our little share of duty in that department of civilization which is represented by country roads. The cyclist goes flying over the highways and bye ways in all sorts of weather, and in the grey dawn or the deepening twilight. If the roads are smooth, dry, and quite free from ruts, not only are his safety and pleasure thereby secured, but the safety of other people is also conserved. When the middle of the road is impassable the cyclist must seek the side. But in the country where there are no pavements for foot passengers the two sides of the highway properly belong to the pedestrians. What is to be done when the rash cyclist or the still more resistless charioteer in the butcher's cart is compelled by its impassibility to leave the crown of the road, and comes careering along the space normally devoted to bipeds? These latter must step into the gutter, or into the hedge where there is no gutter, or be knocked down and perhaps maimed in default. Is there then no "rule of the road" in the country? Certainly there is, but the cyclist or the coachman who is compelled to leave the middle of the road on account of its impassability is as likely to be following the foot passenger as meeting him; or, what is still worse, there may be two cyclists, or two furies in butcher's carts, travelling in opposite directions, one on each side of the road. What is the unlucky foot passenger to do then? If he cannot promptly step into the gutter, or clear the fence, he is more than likely to be promptly knocked down, to the imminent danger of limb or even life.

All philosophic historians have recognised the important part played by roads in the onward progress of peoples. Bad roads mean not only difficult transit but small traffic. They mean limited intercourse between different parts of the country. They mean low prices for provincial exports and high prices for provincial imports. They often mean highwaymen and footpads, or, at the very least, impudent tramps and daring beggars. To a wealthy, æsthetic, and travelling generation like our own they mean the practical closing up of beautiful scenery, lovely parks, charming country houses, and all the simple but dear delights of verdant spring and glorious summer. Clearly, therefore, the "road" problem is one which cannot be left to solve itself or to be made insoluble by the unwilling or incompetent hands of poor farmers or poorer proprietors of the soil. The question belongs to the whole country at large; and the whole country must accept its just responsibility.

It is neither unjust nor unkind to throw upon landlords and farmers the whole blame for the present bad state of many of the country roads and for the miserable muddle into which highway administration generally is fast falling. The simple truth of the matter is that local bodies have been too parsimonious to spend sufficient money themselves on the roads, and too timid to hand them over to central authority. Nobody who knows the influence that landed property has in the counsels of the nation will doubt for a moment that if landlords and tenants had agreed to hand over highway administration to existing or specially constituted central authorities they would have got their wish in the matter long ago. But they preferred to keep both the power and the purse in their own hands, with a result which satisfies neither them nor anybody else. According to Mr. Urban Smith, surveyor of highways for the County of Herts, and author of our pamphlet, "The roads throughout the country are not being maintained in a thoroughly efficient condition . . . . Taking them as a whole it is within the mark to say, that while their condition is such as to cause much undue wear and tear and discomfort, more money is being expended upon them than would, under a proper system, serve to maintain good roads." This proposition, that ill-kept and broken-down roads cost more than well-kept ones, is in accordance with universal experience. A man who has neglected his health until it is thoroughly impaired pays thrice as much for medical attendance and physic as he who, by consulting the doctor betimes, keeps himself always in full vigour. Similarly a half-starved horse or cow eats much more ravenously and devours more food than an animal that is always well fed and thriving.

This common-place and every-day question which we have been discussing shows unmistakably that even in a country of so much robust individualism as we see in our own, a strong central government is imperative. If the most cultured and wealthy of country residents will not be at the trouble and expense of doing the elementary work of making and repairing roads, what would they do if the responsibility of all kinds of public affairs were laid upon their shoulders? It is clear that local authorities must be taught, spurred, reined in, and generally directed by an intelligent and robust central brain and heart. Mr. Urban Smith finds our roads in many places going from bad to worse, and this in spite of our increasing wealth and progressive civilization. Being somewhat of a philosopher he cannot contemplate this partial relapse into barbarism without a kind of horror. Highwaymen and footpads, coaches plunged into ruts two feet deep and incapable of extraction, the smaller bridges all broken down, and the narrower and gutterless lanes converted into running streams; the Squire and his wife returning from a dinner party overturned in their carriage and drowned between the tall hedges that separate the lane from the winter wheat fields—these and the like are the visions that seem to haunt the dreams of the civilized and progressive engineer of the County of Herts.

There is consolation in the thought both for country and town people that matters are likely to be mended. The old-fashioned "Highway Master" and the new-fashioned "Way Warden" have been both alike proved failures. They have been too much under the thumb of the landed proprietor and his tenants; more especially, perhaps, of the latter. Mr. Urban Smith is for bowing the Way Warden out, for taking the branch as well as the main roads entirely out of the hands of the local authorities, and for making their management a department of the work of the County Council. The administrative portion of the work he would commit to thoroughly trained and qualified surveyors, whose status and emoluments should be such that they would be entirely independent both of local magnates and of ignorant and penurious tenant farmers. Something of this kind is so manifestly what ought to be done, that one cannot conceive of anybody, who understands the subject, making the least opposition to it. The result of such a change would no doubt be exactly what Mr. Urban Smith anticipates. All the country roads would be well kept and the cost of their maintenance would be diminished. Not only so but the landlords and tenant farmers would no longer grudge to see money spent on the local roads when it did not come out of the local but out of the county rates. On the contrary, every one of them would be anxious to have the deepest dip into the general purse for the improvement of the roads of his own particular neighbourhood.

